# Experiences with health care and health-related quality of life of patients with hematologic malignancies in Mexico

**DOI:** 10.1186/s12913-020-05498-7

**Published:** 2020-07-10

**Authors:** Svetlana V. Doubova, Eduardo Terreros-Muñoz, Nancy Delgado-Lòpez, Efreen Horacio Montaño-Figueroa, Claudia Infante-Castañeda, Ricardo Pérez-Cuevas

**Affiliations:** 1 Epidemiology and Health Services Research Unit CMN Siglo XXI, Mexican Institute of Social Security, Av. Cuauhtemoc 330, Col. Doctores, Del. Cuauhtemoc, CP 06720 Mexico City, Mexico; 2 Servicio de Hematología, Hospital de Especialidades, Centro Médico Nacional Siglo XXI, Instituto Mexicano del Seguro Social, Ciudad de México, Mexico; 3 Departamento de Hematología, Hospital General de México “Dr. Eduardo Liceaga”. Secretaría de Salud, Ciudad de México, Mexico; 4 Instituto de Investigaciones Sociales, Universidad Nacional Autónoma de México, Ciudad de México, Mexico; 5 Division of Social Protection and Health, Jamaica Country Office, Interamerican Development Bank, Kingston, Jamaica

**Keywords:** Experiences with health care, Health-related quality of life, Hematologic malignancies, Mexico

## Abstract

**Background:**

In Mexico, patients with hematologic malignancies (HMs) are characterized by being at high risk and advanced stages at diagnosis and by having a low cure rate; yet information on their experiences with health care and health-related quality of life (HRQL) is scarce. We aimed to evaluate experiences with health care and HRQL of patients with HMs and the association between these patient-reported measures.

**Methods:**

We conducted a cross-sectional survey in two public oncology hospitals in Mexico City. The study included outpatient cancer patients aged ≥18 years with a diagnosis of leukemia, lymphoma, or multiple myeloma. We used a patient-centered quality of cancer care questionnaire to assess patient experiences with receiving 1) timely care; 2) clear information; 3) information for treatment decision-making; 4) care to address biopsychosocial needs; and 5) respectful and coordinated care. We applied the European Organisation for Research and Treatment of Cancer Quality of Life Questionnaire (EORTC QLQ-C30) to measure HRQL. We performed a multiple linear regression to evaluate the association between patient-reported experiences (independent variables) and the QLQ-C30 summary score (dependent variable).

**Results:**

Of the 515 participating HM patients, 46.6% had lymphoma, 34% leukemia, and 19.4% multiple myeloma; 70.9% were at advanced stages or at high risk. Additionally, 15.1% had anxiety and 12.8% had depression. Over one third (35.9%) reported receiving clear information, 28.5% timely care, 20.6% information for treatment decision-making, 23.7% care that addressed their biopsychosocial needs, and 31% respectful and coordinated care. The mean QLQ-C30 summary score was 71.9 points. Timely care, clear information, and care that addresses biopsychosocial needs were associated with higher HRQL.

**Conclusions:**

Health care services for HM patients at public oncology hospitals in Mexico need improvement. Notably, providing timely care, clear information, and care that addresses patients’ biopsychosocial needs can increase the likelihood of better HRQL. Health care providers should measure and improve the experiences of HM patients with health care.

## Background

Hematologic malignancies are among the ten most common cancers in Latin America and Caribbean countries [1, 2]. In Mexico, hematologic malignancies accounted for 7% of all cancers and 9.8% of cancer deaths in adults ≥20 years of age in 2018 [1]. Compared to high-income countries, patients with hematologic malignancies in Mexico are characterized by being at high risk and advanced stages at diagnosis and by having a lower cure rate [2–6]. Hematologic malignancies negatively affect the quality of life and wellbeing of patients. Living with these diseases triggers physical, emotional, cognitive, social functioning and financial problems [7–9]. Nonetheless, few studies worldwide have addressed the health-related quality of life (HRQL) of patients with hematologic malignancies [10].

Mainstream health care is increasingly moving from treatment focused on the disease to person-centered care [11]. This is a person-oriented model that aims at meeting patient needs, expectations, and preferences through personalized health care that takes their viewpoints into account in the design, provision, and evaluation of services [11]. Person-centered care has the potential to improve health care utilization, quality of care, users’ self-management, self-rated health, and satisfaction, while reducing costs [12–15].

Incorporating patients’ needs, expectations, and preferences in health care requires evaluating their perceptions of care through patient-reported experience measures (PREMs) and their views on their health status through patient-reported outcome measures (PROMs) [16]. PREMs and PROMs are complementary to process of care indicators that inform health system policy and improvement initiatives on quality of care and its impact on patient perceptions of their health and wellbeing [17].

Studies from high-income countries have found deficiencies associated with the health care experiences and health outcomes of patients with hematologic malignancies [7–9]. Research on patient-centered communication reveals insufficient exchange of information between provider and patient, treatment goal misalignments, and discordant role preferences in treatment decision-making [18]. In addition, there is a significant association between patients’ poorer experiences with care and higher symptom burden [19], while having better experiences is associated with improvements in satisfaction and HRQL [14, 20]. However, the information on PREMs and PROMs of patients with hematologic malignancies is limited in Mexico and other Latin America and Caribbean countries. Few studies have reported the effect of hematologic malignancies on patients’ HRQL [21–23] and to the best of our knowledge, there have not been any studies from the Latin America and Caribbean region investigating care of patients with hematologic malignancies from their perspective or the association between patient experiences and HRQL.

Previous research has assessed only the association between demographic and clinical characteristics and HRQL of patients with hematologic malignancies, showing that patients who are women; employed; lacking in social support; at advanced stages or higher risk; or who are experiencing treatment side effects, anxiety, or depression have higher likelihood of lower HRQL [23–25].

Therefore, the objective of the present study is to evaluate experiences with care and HRQL of patients with hematologic malignancies in public health institutions in Mexico and the association between these patient-reported measures.

## Methods

We conducted a cross-sectional survey between April 2018 and September 2019 with patients in two of the largest oncology hospitals in Mexico City—one belonging to the Ministry of Health and the other to the Mexican Institute of Social Security (IMSS)—selected by convenience sampling. Up to 90% of the Mexican population receives health care at either Ministry of Health or IMSS facilities. The IMSS health network provides health insurance to formal sector workers and their families, covering 65 million people [26]. The Ministry of Health provides health care to 54 million people without social security through local health secretariats located in every Mexican state.

The study population comprised outpatient cancer patients aged ≥18 years with one of three hematologic malignancies: lymphoma, acute leukemia, or multiple myeloma. We included patients with at least one hospitalization during the last year, at 5 years or less since diagnosis, and without mental impairment. Two fieldwork-trained nurses interviewed patients after their medical consultations if they met the inclusion criteria, agreed to participate, and signed the informed consent forms. Two field coordinators verified the diagnosis and treatment in patients’ health records.

### Study variables

We used a patient-centered quality of cancer care questionnaire previously validated in Mexico to evaluate experiences with health care [27]. This questionnaire has 30 items in five domains: 1) timely care; 2) clear information; 3) information for treatment decision-making; 4) care to address biopsychosocial needs; and 5) respectful and coordinated care. Each item has a 4-point Likert response option (1 = totally agree, 2 = agree, 3 = disagree, and 4 = totally disagree with the statement about one’s experience in a specific health care encounter). The score for each domain was calculated by reversing the response options, adding all subscale items, and dividing them by the number of items in each subscale/domain for a minimum score of one and a maximum of four per domain [27].

The study outcome variable was perceived HRQL of patients with hematologic malignancies and was measured using the European Organisation for Research and Treatment of Cancer Quality of Life Questionnaire Core 30 (EORTC QLQ-C30) [28]. The EORTC QLQ-30 consists of 30 items grouped into one global health subscale; five function subscales (physical, role, emotional, cognitive, and social functioning); three symptom subscales (fatigue, pain, nausea or vomiting); and six single items covering individual symptoms or problems (shortness of breath, loss of appetite, insomnia, constipation, diarrhea, and financial difficulties). Each item has a 4-point Likert response option scale and two global health questions have a 7-point response option scale. We transformed each subscale linearly to a score of 0–100 with 100 being the best in overall health, functional status, or major symptoms. We used the QLQ-C30 summary score to assess the association between patients’ experience with care and HRQL. This summary score encompasses all function and symptom domains assessed by the EORTC QLQ-C30; it is the mean of 13 QLQ-C30 domain scores, with a higher summary score reflecting better health. The QLQ-C30 summary score has been recommended as a meaningful and reliable measure for oncological research and it has shown greater prognostic value for overall survival than the global HRQL, physical functioning, or any other scale within the QLQ-C30 [29]. It was previously validated in patients with hematologic malignancies [30, 31].

Other study covariates included patient sociodemographic characteristics (gender, age, educational attainment, and marital status) and clinical history (time since diagnosis, cancer type and stage, anxiety, and depression). We categorized the following variables: patient age (≤45; 46 to 64; ≥65 years); educational attainment (completed elementary school or less; secondary school; high school or higher); cancer type (leukemia, lymphoma, or multiple myeloma); cancer stage or risk (early stage [I–II] or low and standard risk; advanced stage [III–IV] or high and very high risk); time since diagnosis (≤6 months; 7 to 12 months; >1 to 5 years). We measured anxiety and depression using the Hospital Anxiety and Depression Scale composed of 14 items previously validated in Spanish with cancer patients [32]. Each item has a 4-point Likert scale response that ranges from 0 to 3. A summary score of ≥11 points in each domain indicates anxiety or depression [33].

### Sample size and statistical analysis

We secured a minimum of 10 participants per covariate in the multiple regression analysis [34].

We performed descriptive and exploratory analyses and found that the QLQ-C30 summary score had a normal distribution; however, the domains of perceived experiences with patient-centered cancer care had a skewed distribution. Therefore, to determine the association between independent and dependent variables, we dichotomized the independent variables as high and low patient-centered quality of cancer care by using the 75th percentile as a cut-off value. The distribution of variables supported this decision, including the low frequency of patients at the 85th, 90th, and 95th percentiles. We built five high patient-centered quality of cancer care variables: 1) timely care = 4.0 points; 2) clear information = 4.0 points; 3) information for treatment decision-making ≥3.6 points; 4) care for biopsychosocial needs ≥2.08 points; and 5) respectful and coordinated care ≥3.83 points.

We performed the Student t-test for two-group comparisons and one-way analysis of variance for the difference in means among more than two groups to compare HRQL by patients’ sociodemographic characteristics, clinical characteristics, and patient-centered quality of cancer care domains.

As recommended by VanderWeele [35], we modeled a multiple regression analysis with simultaneous inclusion of all conceptually and clinically relevant variables to determine the independent association between patients’ experiences as measured by the patient-centered quality of cancer care domains and HRQL independent from other covariates. We performed a bootstrapped linear regression model with 10,000 bootstrap replications. The bootstrap method has a less restrictive assumption about the sample being representative of the population, making it a large sample method akin to the Central Limit Theorem [36, 37]. In addition, we considered the cluster effect. Given that the study included patients from IMSS and Ministry of Health hospitals, one of the assumptions was that the measurements within each hospital may not be independent because patients treated in the same hospital were more likely to receive similar quality of care than patients from other hospitals. Thus, we adjusted the standard errors by computing clustered robust standard errors for the coefficients.

Stata 14.0 (Stata Corp, College Station, TX, USA) was used for the analysis; *p* < 0.05 was considered to be statistically significant.

### Ethics considerations

The study was approved by the IMSS National Research and Ethics Committee (registry number R-2017-785-042).

## Results

Nearly 90% of eligible patients with hematologic malignancies agreed to participate. The main reasons for not participating were lack of interest in answering the survey; lack of time; and fatigue, weakness, or pain. A total of 515 patients with hematologic malignancies participated in the study (Table 1). Half of the participants were women (50.9%) and the average age was 48.6 years; 59.6% had completed elementary school or secondary school and the rest had completed high school. Most participants were married (60.8%) and received health care at the IMSS hospital (63.7%).

**Table 1:** Characteristics of study participants (*n* = 515)

Variable	n (%)
**Sociodemographic characteristics**	
Women	262 (50.9)
Age, mean (SD)	48.6 (17.6)
≤ 45 years	206 (40.0)
46 and < 65 years	202 (39.2)
≥ 65 years	107 (20.8)
Education	
Elementary school or less	151 (29.3)
Secondary school	156 (30.3)
High-school or higher	208 (40.4)
Married or free union	313 (60.8)
Hospital	
IMSS	328 (63.7)
Ministry of Health	187 (36.3)
**Type of cancer**	
Lymphoma^a^	240 (46.6)
Leukemia^b^	175 (34.0)
Multiple myeloma	100 (19.4)
Time since cancer diagnosis	
≤ 6 months	257 (49.9)
7–12 months	194 (37.7)
> 1 and ≤ 5 years	64 (12.4)
Advanced stages (III-IV)/High risk	365 (70.9)
Anxiety	78 (15.1)
Depression	66 (12.8)

^a^Lymphoma no Hodgkin: 173 participants (33.6%); Lymphoma Hodgkin: 67 participants (13.0%); ^b^Acute lymphocytic leukemia: 111 participants (21.6%); Acute myelogenous leukemia: 64 participants (12.4%)

The most frequent hematologic malignancy was lymphoma (46.6%) (non-Hodgkin lymphoma (33.6%) and Hodgkin lymphoma (13.0%)); followed by leukemia (34%), which comprised acute lymphocytic leukemia (21.6%) and acute myelogenous leukemia (12.4%); and multiple myeloma (19.4%). Most participants had been diagnosed with cancer < 6 months prior (49.9%) or between 7 and 12 months prior (37.7%) and were at advanced stages (III–IV) or at high risk (70.9%). Furthermore, 15.1% had anxiety and 12.8% had depression.

The respectful and coordinated care domain got the highest patient-centered quality of cancer care score on the 4-point scale (3.5 points); care to address biopsychosocial needs got the lowest patient-centered quality of cancer care score (1.5 points). Based on the 75th percentile, only one third (35.9%) reported receiving clear information, 28.5% timely care, 20.6% information for treatment decision-making, 23.7% care that addressed their biopsychosocial needs, and 31.8% respectful and coordinated care (Table 2).

**Table 2:** Perceived quality of patient-centered cancer care and quality of life of patients with hematologic malignancies (*n* = 515)

	Median (percentile 25th, 75th)
**Perceived quality of patient-centered cancer care**	
Timely care	3.0 (2.33, 4.00)
Clear information	3.3 (3.0, 4.00)
Information for treatment decision-making	2.8 (2.0, 3.60)
Addressing biopsychosocial needs	1.5 (1.1, 2.08)
Respectful and coordinated care	3.5 (3.0, 3.83)
**High quality of patient-centered cancer care** **(≥75th percentile)**	%
Timely care	147 (28.5)
Clear information	185 (35.9)
Information for treatment decision-making	106 (20.6)
Addressing biopsychosocial needs	122 (23.7)
Respectful and coordinated care	164 (31.8)
**Perceived quality of life**	Mean (SD)
*Global Health Status*	59.2 (19.3)
Physical functioning	65.0 (27.1)
Role functioning	51.7 (36.7)
Emotional functioning	69.8 (27.7)
Cognitive functioning	76.6 (25.9)
Social functioning	60.0 (32.8)
*Symptom scales*	
Fatigue	40.1 (29.3)
Nauseas and vomiting	13.9 (23.0)
Pain	28.3 (30.8)
Dyspnea	18.8 (28.0)
Insomnia	33.1 (35.9)
Appetite loss	18.2 (28.6)
Constipation	24.9 (32.8)
Diarrhea	10.7 (21.9)
Financial difficulties	49.7 (38.1)
**HRQL Summary Score**^a^	71.9 (17.9)

^a^HRQL Summary Score: EORTC QLQ-C30 Summary Score

With regards to HRQL, the mean score for global health was 59.2 points on a scale of 0–100. Cognitive functioning had the highest HRQL score (mean 76.6) and role functioning had the lowest score (mean 51.7). Significant symptoms or problems were related to financial difficulties (mean 49.7), fatigue (mean 40.1), and insomnia (mean 33.1). The mean for the HRQL summary score was 71.9 points (Table 2).

Table 3 depicts the HRQL summary score of patients with hematologic malignancies by their general, clinical, and patient-centered quality of cancer care characteristics. Patients 45 years of age or younger and those who were single reported higher HRQL than their older and married counterparts. There were no statistically significant differences in HRQL summary scores between men and women, patients with a different educational background, or those treated at IMSS vs. Ministry of Health. With regards to clinical or cancer care characteristics, patients diagnosed with leukemia, those diagnosed with hematologic malignancy between 7 and 12 months prior, and those without anxiety or depression reported higher HRQL summary scores than their counterparts. Furthermore, patients who experienced receiving timely care, clear information, and care that addressed their biopsychosocial needs perceived having higher HRQL summary scores than those who did not.

**Table 3:** Quality of life by patients’ characteristics and experiences with cancer care (*n* = 515)

	HRQL Summary Score^a^
	Mean (SD)
Sex	
Man	72.4 (18.3)
Women	71.4 (17.5)
Age****	
≤ 45 years	76.0 (16.1)
46 and < 65 years	69.3 (19.0)
≥ 65 years	69.0 (17.7)
Education	
Elementary school or less	70.5 (17.8)
Secondary school	71.1 (19.1)
High-school degree or higher	73.6 (16.9)
Marital status**	
Married or free union	70.3 (18.6)
Single, divorced or widowed	74.5 (16.4)
Hospital	
Social Security	70.9 (18.8)
Ministry of Health	73.8 (15.9)
Clinical history	
Type of cancer****	
Leukemia	78.5 (13.6)
Lymphoma	70.6 (18.7)
Multiple myeloma	63.6 (18.2)
Cancer stage/risk	
Early stages (I-II)/Low/standard risk	71.7 (16.9)
Advanced stages (III-IV)/High risk	71.9 (18.2)
Time since cancer diagnosis**	
≤ 6 months	69.8 (18.5)
7–12 months	74.7 (16.5)
>1 and 5 years	71.8 (18.4)
Anxiety****	
No	75.5 (15.6)
Yes	52.1 (16.9)
Depression****	
No	74.7 (16.4)
Yes	52.8 (15.5)
**High quality of patient-centered cancer care (≥75th percentile)**	
Timely care*	
Yes	74.6 (18.3)
No	70.9 (17.6)
Clear information***	
Yes	75.8 (16.6)
No	69.7 (18.2)
Information for treatment decision-making	
Yes	74.9 (18.0)
No	71.1 (17.8)
Addressing biopsychosocial needs****	
Yes	79.6 (13.1)
No	69.5 (18.5)
Respectful and coordinated care	
Yes	72.5 (18.5)
No	71.6 (17.6)

*p < 0.05; **p < 0.01; ***p < 0.001; ****p < 0.000. ^a^HRQL Summary Score: EORTC QLQ-C30 Summary Score

Receiving timely care, clear information, and care that addressed patient biopsychosocial needs were patient-reported experiences associated with higher HRQL summary scores after adjusting for patient sociodemographic and clinical characteristics (Table 4). Specifically, timely care was associated with a 1.34 point increase in HRQL (95%CI: 0.52; 2.16), clear information was associated with a 5.14 point increase (95%CI: 4.88; 5.40), and care that addressed patient biopsychosocial needs was associated with a 4.39 point increase (95%CI: 1.87; 6.91).

**Table 4:** Experiences with cancer care associated with higher quality of life^a^ of patients with hematologic malignancies (*n* = 515)

Variables	Adjusted ß (95%CI), p
**Perceived high quality of patient-centered cancer care**^b^	
Timely care	**1.34 (0.52; 2.16), 0.001**
Clear information	**5.14 (4.88; 5.40), 0.000**
Information for treatment decision-making	−2.09 (− 5.08; 0.90), 0.170
Addressing biopsychosocial needs	**4.39 (1.87; 6.91), 0.001**
Respectful and coordinated care	−3.21 (−7.82; 1.40), 0.172
**Covariates used to adjust the model**	
Sociodemographic factors	
Women^b^	0.94 (0.88; 2.75), 0.311
≤ 45 years of age^b^	**3.51 (3.30; 3.72), 0.000**
46 and < 65 years	**2.05 (1.78; 2.33), 0.000**
Elementary school or less^b^	0.77 (−0.65; 2.19), 0.286
Secondary school	−0.62 (−2.50; 1.26), 0.519
Single, divorced or widowed^b^	1.48 (−1.08; 4.05), 0.257
Clinical factors	
Lymphoma^b^	**−3.48 (−6.94; −0.02), 0.049**
Multiple Myeloma^b^	**−10.01 (− 12.63; −7.38), 0.000**
Advanced stages (III-IV)/High risk^b^	0.87 (−2.90; 4.64), 0.652
≤ 6 months since cancer diagnosis^b^	−2.28 (−6.44; 1.89), 0.284
7–12 months since cancer diagnosis^b^	−0.28 (− 2.37; 1.82), 0.795
Anxiety^b^	**− 17.21 (− 19.54; − 14.87), 0.000**
Depression^b^	**−12.06 (− 15.14; −8.98), 0.000**

Abbreviations: *95%CI* 95% Confidence Interval. ^a^Quality of life measured by the EORTC QLQ-C30 Summary Score. ^b^Reference categories: low quality of patient-centered cancer care, men; age 65 years of age or older; high-school or higher; married; leukemia; early stages/low/or standard risk; time since diagnosis > 1 and ≤ 5 years; without anxiety or depression. The bold values highlight the statistically significant adjusted ß

## Discussion

Patients’ experiences with care and their HRQL are pivotal patient-reported indicators of health care quality. Our study revealed critical deficiencies in cancer care experiences of patients with hematologic malignancies in two public health institutions in Mexico. Less than a third of patients with hematologic malignancies felt they experienced timely care, had information for treatment decision-making, and had their biopsychosocial needs addressed. The HRQL summary score of patients in this study was lower than that of patients with hematologic malignancies from high-income countries [29–31]. Additionally, timely care, clear information, and care that addressed biopsychosocial needs of patients with hematologic malignancies were associated with higher HRQL.

HRQL is the most informative and frequently used PROM in oncology. It is a multidimensional concept referring to how patients perceive the impact of their disease and treatment on the physical, psychological, and social aspects of their lives [38]. Research has shown the prognostic value of HRQL scores and their usefulness for the treatment decision-making of cancer patients [39]. EORTC QLQ-C30 is a robust scale for measuring HRQL [28] and its summary score has been identified as a meaningful and reliable measure for oncological research [29]. Our study found that the HRQL summary score of participating patients with hematologic malignancies was generally lower when compared with scores from high-income countries [29–31]. For instance, in the Netherlands [31], the average HRQL summary score of patients with hematologic malignancies varied from 87.2 points for Hodgkin lymphoma to 75.4 points for multiple myeloma, while the average HRQL summary score of our study population was 71.9, varying from 78.5 points for leukemia to 63.3 points for multiple myeloma. However, data on EORTC QLQ-C30 summary scores of patients with hematologic malignancies in low- and middle-income countries and Latin America is still unavailable.

In our study, only one out of three patients with hematologic malignancies reported experiencing high patient-centered quality of cancer care. Delivering high-quality cancer care is challenging in low- and middle-income countries due to shortages in specialized human resources, such as oncologists/hematologists, pathologists, radiologists, and transplantolologists of haematopoietic progenitor cells, among others. There are also financial constraints and rising costs of chemotherapy agents, radiation therapy, and imaging tests. These shortages might negatively affect patients’ experiences with care. Nonetheless, patient-centered cancer care that integrates respectful, coordinated, and timely care; provides clear information; and addresses patients’ health-related needs should be delivered to all patients, including in resource-limited settings [40].

Shared decision-making has been associated with improved patient-provider relationships, patient treatment adherence, satisfaction with care, and better health outcomes [41–45]. Clear information about the effectiveness of treatment options and possible side effects is a prerequisite for shared decision-making in clinical practice. However, patients frequently perceive a lack of information. In a study from Australia, only 23% of patients with hematologic malignancies reported that doctors had explained how each treatment option might affect their life expectancy [46]. One study from Guadalajara City in Mexico also identified insufficient information as an important gap in health care for cancer patients at IMSS [47]. In our study, 80% of patients with hematologic malignancies perceived a lack of clear information, especially for treatment decision-making. Patient-centered health education should be promoted as a strategy for delivering information to patients, including through a combination of printed materials, audiovisual aids, interactive media, and personal counseling [48].

Patients with hematologic malignancies face multiple complex biopsychosocial needs, including physical, emotional, and social needs, such as how to organize day-to-day tasks and leisure activities and how to find other cancer patients to talk to about their experiences [49, 50]. At the same time, these needs are not identified as a priority in clinical care and hence, they are often unaddressed by health care providers. In Australia, lack of emotional support and information about how to connect with other cancer patients was the most commonly identified unmet need of patients with hematologic malignancies [46]. A previous study in patients with solid cancers at IMSS found a high prevalence of patients with unmet physical/daily living and psychological needs [51]. This study identified that only 23% of patients perceived that their biopsychosocial needs were being addressed. In addition, our study found that receiving clear information and care that addressed patient biopsychosocial needs were associated with statistically significant 4–5 point increases in HRQL of patients, which is also clinically relevant [52]. This result is in line with a United States study which found that physicians’ involvement in helping patients with hematologic malignancies adjust to their physical and emotional challenges was positively associated with patients’ HRQL [20].

This study has several strengths and limitations. The strengths include the use of validated questionnaires to measure patients’ experiences with health care and HRQL and the focus on patients with hematologic malignancies that are currently underrepresented among PREMs and PROMs studies. The study’s limitations comprise the cross-sectional study design, which does not allow for making causal inferences or identifying the direction of association between study variables. Additionally, the sample included only patients from two public oncology hospitals in Mexico City. Thus, the study results cannot be generalized to private oncology hospitals or public oncology hospitals in other Mexican states.

## Conclusions and study implications

Health care services for patients with hematologic malignancies at IMSS and Ministry of Health facilities need to be improved. Notably, patient-centered cancer care that is timely, provides clear information and addresses patients’ biopsychosocial needs can increase the likelihood of better HRQL.

### The study results have several clinical and research implications

First, the study highlights the importance of measuring and reporting patients’ experiences and outcomes to provide insights into gaps in patient-centered cancer care and guide improvement strategies to enhance the health outcomes of this population. This is especially crucial given that the evaluation of cancer patients’ experiences and outcomes is not currently a standard practice in Latin American countries. The assessment of cancer patients’ reported experiences and outcomes should include both public and private health care facilities due to possible differences in care between these providers and quality of life of their respective patients. Second, although we cannot make causal inferences from our observational study, the results indicate a need for further research aimed at designing and evaluating patient-centered quality improvement programs to increase patients’ HRQL. Finally, similar to other studies [53], our results suggest that a multidisciplinary approach to cancer care is needed to effectively respond to the complex biopsychosocial needs experienced by the growing number of patients with hematologic malignancies and their families. Such an approach should be strengthened by the provision of timely care and clear information.

## Data Availability

Data is available from the first author upon reasonable request.

## Abbreviations

EORTC QLQ-30: Quality of life questionnaire of the European Organisation for Research and Treatment of Cancer
HRQL: Health-related quality of life
IMSS: Mexican Institute of Social Security
PREMs: Patient-reported experience measures
PROMs: Patient-reported outcome measures
